# Endoscopic Ultrasound vs. Computed Tomography for Gastric Cancer Staging: A Network Meta-Analysis

**DOI:** 10.3390/diagnostics11010134

**Published:** 2021-01-16

**Authors:** Bogdan Silviu Ungureanu, Victor Mihai Sacerdotianu, Adina Turcu-Stiolica, Irina Mihaela Cazacu, Adrian Saftoiu

**Affiliations:** 1 Gastroenterology Department, University of Medicine and Pharmacy of Craiova, 200349 Craiova, Romania; bogdan.ungureanu@umfcv.ro (B.S.U.); sacerdotianumihai@gmail.com (V.M.S.); adriansaftoiu@gmail.com (A.S.); 2 Pharmacoeconomics Department, University of Medicine and Pharmacy of Craiova, 200349 Craiova, Romania; 3 Oncology Department, Fundeni Clinical Hospital Bucharest, 022328 Bucharest, Romania; irina.cazacu89@gmail.com

**Keywords:** endoscopic ultrasound, computed tomography, gastric cancer staging

## Abstract

Gastric cancer preoperative staging is of outmost importance to assure proper management of the disease. Providing a relevant clinical stage relies on different imaging methods such as computed tomography (CT) or endoscopic ultrasound (EUS). We aimed to perform a network meta-analysis for gastric cancer clinical stage diagnostic tests, thus comparing the diagnostic accuracy of EUS vs. multidetector CT (MDCT) and EUS vs. EUS + MDCT. We plotted study estimates of pooled sensitivity and specificity on forest plots and summary receiver operating characteristic space to explore between-study variation in the performance of EUS, MDCT and EUS + MDCT for T1–T4, N0–N3, M0–M1 when data were available. Exploratory analyses were undertaken in RevMan 5. We included twelve studies with 2047 patients. Our results suggest that EUS was superior to MDCT in preoperative T1 and N staging. MDCT is more specific for the M stage but no significant difference in sensitivity was obtained. When comparing EUS vs. EUS + MDCT for T1 both sensitivity and specificity were not relevant. No significant differences were observed in T2–T4 stages. Even though EUS helped differentiate between the presence of invaded nodules, N stages should be carefully assessed by both methods since there is not sufficient data.

## 1. Introduction

Accurate preoperative staging for gastric cancer is imperative for the proper management of the disease [1]. While the curative approach still involves tumor excision, choosing the right therapy can be difficult due to clinical staging challenges [1,2]. Early stages can be treated endoscopically or surgically by endoscopic submucosal dissection (ESD), endoscopic mucosal resection, or through laparoscopic surgery, whereas intermediate stages require neoadjuvant chemotherapy to improve tumor status for subsequent resection [3]. Thus, tumor depth invasion and additional malignant lymph nodes assessment are the cornerstones of therapeutic management.

Multimodal imaging using endoscopic ultrasound and computer tomography (CT) should be used for clinical staging. Multidetector computer tomography (MDCT) has overcome some of the drawbacks of the CT-scan and is used for both distant metastasis diagnosis and loco-regional disease. Endoscopic ultrasound (EUS) has been adopted as a useful tool for depth penetration assessment of the gastrointestinal tract, and is generally used for rectal cancer staging as well as esophageal and gastric cancer [4,5].

The use of these imaging techniques to characterize the primary tumor (cT) after the biopsy results is essential for therapeutic management. EUS is relevant for cT assessment, especially for T1a and T1b, where it may provide valuable data for ESD and EMR procedures and also for the N stage where fine needle aspiration may be performed for cytology diagnosis [2]. On the other hand, MDCT results have shown an improved accuracy for identifying locoregional disease [6,7,8]. However, despite the worldwide use of these techniques, some of the results for gastric cancer staging are still debatable. A previous meta-analysis found that EUS may be superior to MDCT in preoperative T1 and N staging. [9]. Thus, our objective was to assess the currently available data on gastric cancer staging involving EUS and MDCT, and to perform a network meta-analysis for diagnostic tests to compare the diagnostic accuracy of EUS vs. MDCT and of EUS vs. EUS + MDCT.

## 2. Materials and Methods

### 2.1. Search Methods

This meta-analysis was conducted in accordance with the PRISMA (Preferred Reporting Items for Systematic Reviews and Meta-Analyses) for Diagnostic Test Accuracy [10]. We performed a thorough literature search for studies reporting the accuracy of EUS and MDCT scans from inception to 15 September 2020. We searched the PubMed and Web of Science and the references of the included studies to identify further studies. In our research we used the following keywords: (“endoscopic ultrasound” OR “EUS”) AND (“multi-detector computed tomography” OR “MDCT” OR “multi-slice spiral computed tomography” OR “MSCT”) AND (“gastric cancer” OR “gastric adenocarcinoma”).

### 2.2. Selection Criteria

The inclusion criteria were: (1) studies reporting cross-sectional information on the index test (endoscopic ultrasonography (EUS) and multidetector-row computed tomography (MDCT)) and the reference standard (confirmation by histopathological analysis of surgical specimens); (2) studies with sufficient data for reporting true-positive (TP), true-negative (TN), false-positive (FP) and false-negative (FN) results; (3) adults with gastric cancer; (4) prospective or retrospective, cross-sectional studies or randomized clinical trials. We accepted the criteria stated by the authors to classify the T and the N staging, which is from the fourth edition to the seventh edition of the TNM classification, and planned to explore it as a source of heterogeneity. We excluded studies of low methodological quality, which may result in arriving at false outcomes. We excluded case series, review articles, abstracts or letters; and studies published in a language other than English.

### 2.3. Data Collection and Analysis

Two review authors (B.S.U. and V.M.S.) independently screened all titles and abstracts yielded from the searches to identify relevant studies according to the aforementioned selection criteria and extracted the data. Any differences between the review authors were arbitrated by a third author (A.T.-S.). The following data from each included study were extracted: first author, year of publication, the total number of patients, TP, TN, FP and FN for every index test (EUS, MDCT and EUS + MDCT), and the edition of TNM classification. The data were extracted for T1, T2, T3, T4, N0, N1, N2, N2, N3, M0, M1 when data were available. We contacted the correspondence study authors if we needed more information (for example, TP, TN, FP, FN for every T1, T2, T3, T4 stage).

### 2.4. Assessment of Methodological Quality

The two review authors (B.S.U. and V.M.S.) independently assessed the quality of studies using the Quality Assessment of Diagnostic Accuracy Studies-2 (QUADAS-2) assessment tool, which evaluates patient selection, the index test, reference standard, and flow and timing. Signaling questions were included to facilitate judgment about applicability which was determined as “low”, “high”, or “unclear”. Any differences between the review authors were arbitrated by a third author (A.T.-S.). All these domains were assessed for risk and bias.

### 2.5. Statistical Analysis

We plotted the study estimates of pooled sensitivity and specificity on forest plots and summary receiver operating characteristic (SROC) space to explore between-study variation in the performance of EUS, MDCT and EUS + MDCT for T1, T2, T3, T4, N0, N1, N2, N2, N3, M0, M1 when data were available, using a bivariate random-effects model and a Bayesian approach. Exploratory analyses were undertaken in Review Manager 5 (RevMan 5. Version 5.4.1, The Cochrane Collaboration, 2020) and we used R for the definitive analyses. The R-package, *mada* was used for the meta-analysis of diagnostic accuracy.

The area under the ROC curve (AUC) was calculated to estimate the overall accuracy. A preferred test has an AUC close to 1, while a poor test has an AUC close to 0.5. 

Indirect comparisons provided useful evidence and our network meta-analysis included indirect evidence with no closed loop. We used mean difference (95% confidence interval) for the comparison of the sensitivity and specificity of the two index tests: EUS vs. MDCT and EUS vs. EUS + MDCT. Heterogeneity was investigated through the Higgins I^2^; a value of 0% indicates no observed heterogeneity, and larger values show increasing heterogeneity. We performed the χ2 test to assess the heterogeneity of sensitivities and specificities, the null hypothesis being, in both cases, that all are equal for all the studies. The random-effects model was performed if there was heterogeneity between studies, otherwise the fixed-effects model was used. The significance level was 0.05.

## 3. Results

### 3.1. Electronic Search Results and Study Characteristics

**Figure 1:**
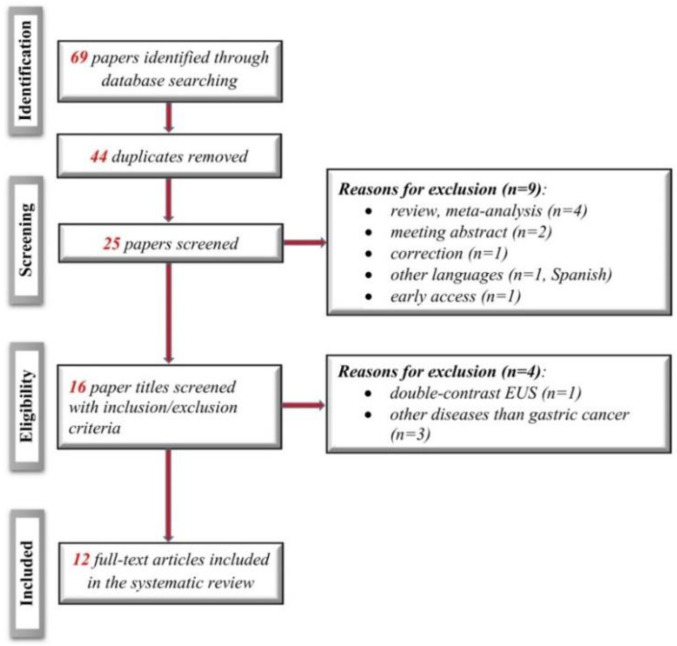
Diagram of the study flow according to the preferred reporting items for systematic reviews and meta-analyses (PRISMA) diagram.

We identified 12 studies according to the search strategy (including four new studies since the previous review). Figure 1 shows the flow diagram for the review process, with the steps according to the PRISMA statement [11].

The characteristics of the included studies are presented in Table 1. The 12 studies involved a total of 1859 patients diagnosed with gastric adenocarcinoma and pre-surgical staging with MDCT and EUS. The majority of patients were male (n = 1302, 70.03%). The most common tumor location was within the antrum/lower part of the stomach.

### 3.2. Quality Assessment of the Included Studies

**Figure 2:**
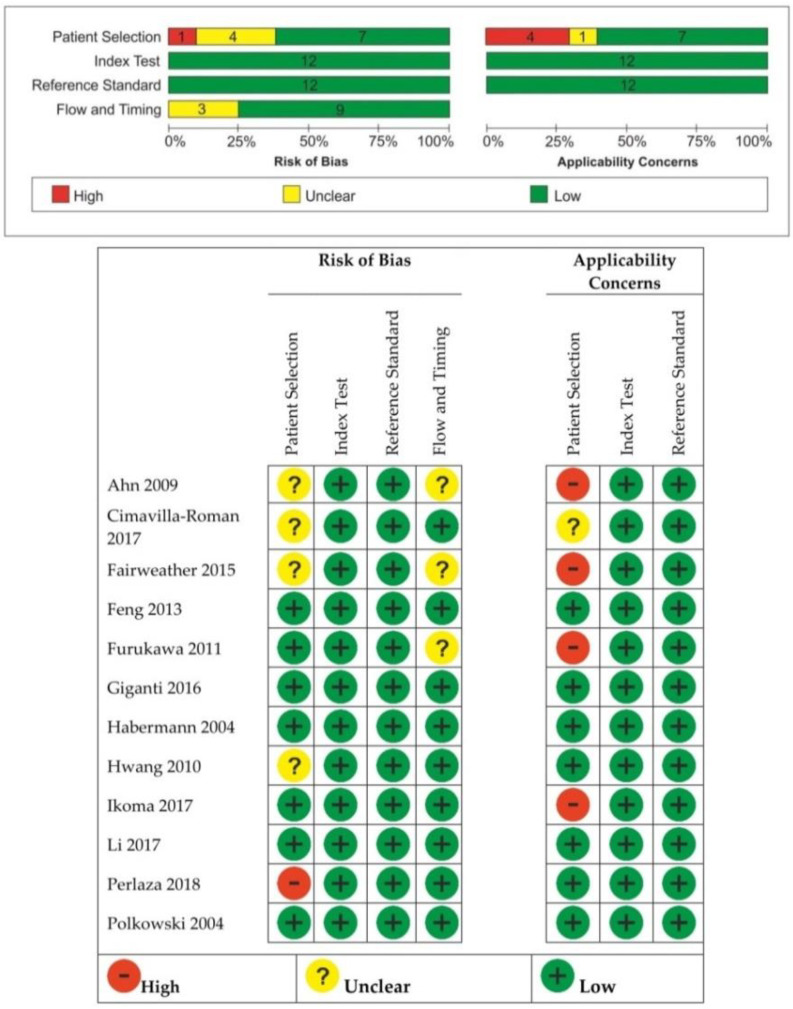
Risk of bias and applicability concerns summary (QUADAS-2).

Risk of bias and applicability concerns are shown in Figure 2. The methodology for patient selection was unclear in four studies [12,13,14,19]. The risk of bias was considered high if patients with early-stage gastric cancer were excluded [21]. The studies where not all of the participants were included in the analysis for both EUS and MDCT were at high risk of bias with regard to the flow and timing domain, and patient selection was an applicability concern [12,14,16,20]. Three studies [12,14,16] were at unclear risk of bias for flow and timing because it was unclear if there was an inappropriate interval between the index test and reference standard.

### 3.3. Data Synthesis

The results are summarized in Table 2 (the overall findings of EUS and MDCT) and Table 3 (the overall findings for EUS and EUS + MDCT).

#### 3.3.1. EUS vs. MDCT

##### T1 Stage

**Figure 3:**
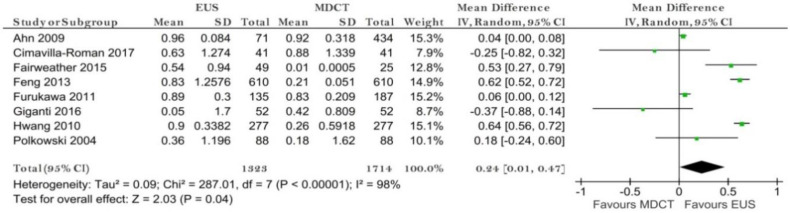
Forest plot of sensitivity for T1 staging.

**Figure 4:**
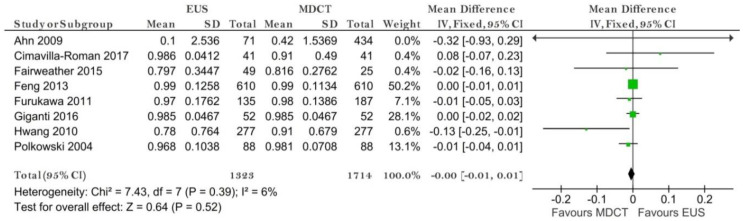
Forest plot of specificity for T1 staging.

Eight studies reporting data on 1714 patients were included for this test, allowing meta-analysis to be performed as in Figure 3 for sensitivity and as in Figure 4 for specificity. The sensitivity value for EUS was significantly higher than for MDCT (*p* = 0.04) after using a random-effects model for the high heterogeneity of the included studies (χ^2^ = 287.01, I^2^ = 98%, *p* < 0.0001).

The specificity value for EUS was smaller than for MDCT, but with no significant difference (*p* = 0.52) after using a fixed-effects model for no significant heterogeneity between studies (χ^2^ = 7.43, I^2^ = 6%, *p* = 0.39).

**Figure 5:**
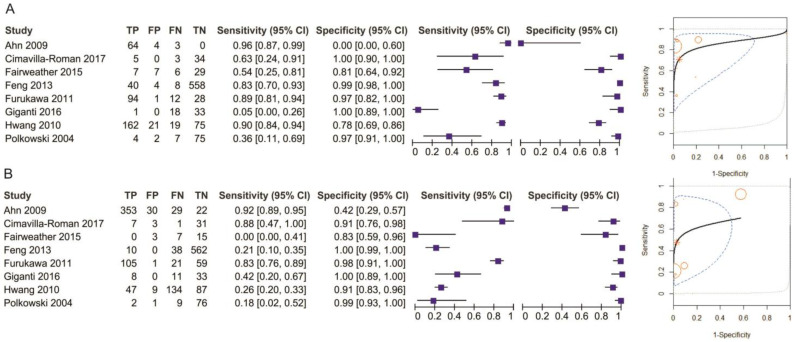
Pooled sensitivity and specificity for T1 staging of EUS (**A**) and MDCT (**B**).

The pooled sensitivity of EUS for the T1 stage was 0.707 (95%CI, 0.433–0.884), higher than that of MDCT, which was 0.519 (95%CI, 0.256–0.772). The pooled specificity value of EUS for the T1 stage was 0.931 (95%CI, 0.755–0.983), slightly smaller than that of MDCT, which was 0.941 (95%CI, 0.798–0.985). The AUC for EUS (0.903) was bigger than for MDCT (0.774) and the summary ROC curve location for T1 invasion using EUS was closer to the upper left corner than those using MDCT, which indicate the better diagnostic performance of EUS vs. MDCT (Figure 5).

##### T2 Stage

Nine studies reporting data on 1374 patients were included for this test, allowing meta-analysis to be performed. The sensitivity value for EUS was slightly higher, but not statistically significant, than that for MDCT (*p* = 0.67) after using a random-effects model for the high heterogeneity of the included studies (χ^2^ = 77.46, I^2^ = 90%, *p* < 0.0001).

The specificity value for EUS was higher than for MDCT, but with no significant difference (*p* = 0.32) after using a random-effects model for the significant heterogeneity between studies (χ^2^ = 19.02, I^2^ = 58%, *p* =0.01).

The pooled sensitivity of EUS for the T2 stage was 0.671 (95%CI, 0.531–0.785), higher than that of MDCT, which was 0.592 (95%CI, 0.396–0.763). The pooled specificity value of EUS for the T2 stage was 0.831 (95%CI, 0.79–0.866), higher than that of MDCT, which was 0.797 (95%CI, 0.732–0.849). The AUC for EUS (0.845) was bigger than for MDCT (0.793) and the summary ROC curve location for T2 invasion using EUS was closer to the upper left corner than those using MDCT, which indicate the better diagnostic performance of EUS vs. MDCT.

##### T3 Stage

Nine studies reporting data on 1374 patients were included for this test. The sensitivity value for EUS was slightly higher, but not statistically significant, than for MDCT (*p* = 0.90) after using a random-effects model for the high heterogeneity of the included studies (χ^2^ = 46.31, I^2^ = 83%, *p* < 0.00001). The specificity value for EUS was slightly higher than for MDCT, but with no significant difference (*p* = 0.25) after using a random-effects model for the significant heterogeneity between studies (χ^2^ = 37.84, I^2^ = 79%, *p* < 0.00001).

The pooled sensitivity of EUS for the T3 stage was 0.638 (95%CI, 0.493–0.762), almost the same as that of MDCT, which was 0.634 (95%CI, 0.405–0.815). The pooled specificity value of EUS for the T3 stage was 0.842 (95%CI, 0.748–0.906), higher than that of MDCT, which was 0.808 (95%CI, 0.677–0.894). The AUC for EUS (0.814) was bigger than for MDCT (0.804) and the summary ROC curve location for T3 invasion using EUS was the same as the upper left corner as those using MDCT, which indicate no difference between the diagnostic performance of EUS vs. MDCT.

##### T4 Stage

Nine studies reporting data on 1374 patients were included for this test. The sensitivity value for EUS was slightly smaller, but not statistically significant, than for MDCT (*p* = 0.59) after using a random-effects model for the high heterogeneity of the included studies (χ^2^ = 50.14, I^2^ = 84%, *p* < 0.00001). The specificity value for EUS was smaller, but not statistically significant, than for MDCT (*p* = 0.38) after using a random-effects model for the significant heterogeneity between studies (χ^2^ = 21.99, I^2^ = 64%, *p* = 0.005).

The pooled sensitivity of EUS for the T4 stage was 0.518 (95%CI, 0.333–0.698), smaller than that of MDCT, which was 0.657 (95%CI, 0.463–0.811). The pooled specificity value of EUS for the T4 stage was 0.947 (95%CI, 0.878–0.978), almost the same as that of MDCT, which was 0.957 (95%CI, 0.914–0.978). The AUC for EUS (0.846) was smaller than for MDCT (0.93) and the summary ROC curve location for T4 invasion using EUS was the same as the upper left corner as those using MDCT, which indicate no difference between the diagnostic performance of EUS vs. MDCT.

##### N Stage (N−/N+)

**Figure 6:**
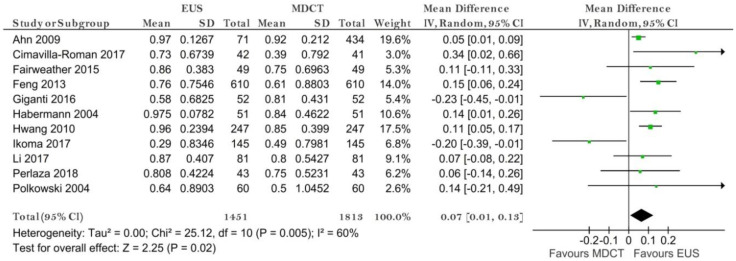
Forest plot of sensitivity for N staging.

Eleven studies reporting data on 1813 patients were included for this test. The sensitivity for EUS was significantly higher than for MDCT (*p* = 0.02) after using a random-effects model (χ^2^ = 25.12, I^2^ = 60%, *p* = 0.005) (Figure 6).

**Figure 7:**
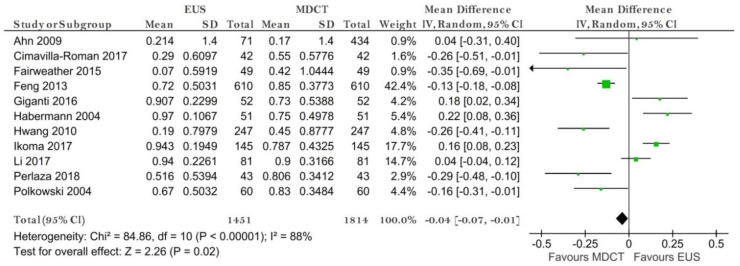
Forest plot of specificity for N staging.

The specificity value for MDCT was significantly higher than for EUS (*p* = 0.02) after using a random-effects model for the significant heterogeneity between studies (χ^2^ = 84.86, I^2^ = 88%, *p* < 0.00001) (Figure 7).

**Figure 8:**
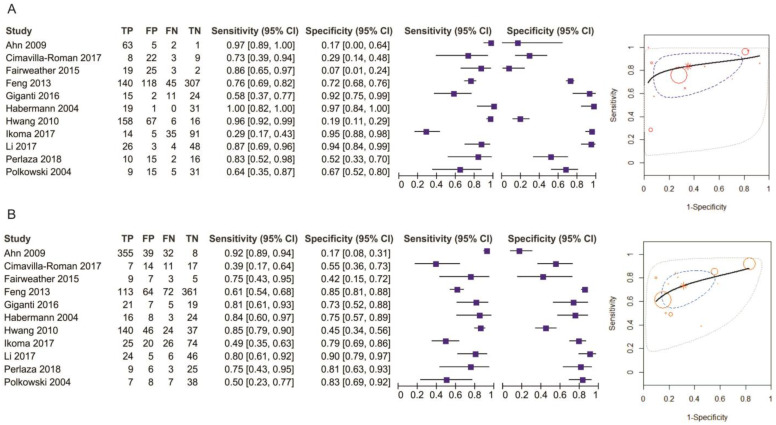
Pooled sensitivity and specificity for N staging of EUS (**A**) and MDCT (**B**).

The pooled sensitivity of EUS for the N- stage was 0.794 (95%CI, 0.644–0.892), higher than that of MDCT, which was 0.726 (95%CI, 0.610–0.819). The pooled specificity value of EUS for the N-stage was 0.636 (95%CI, 0.372–0.837), smaller than that of MDCT, which was 0.681 (95%CI, 0.528–0.804). The AUC for EUS (0.795) was bigger than for MDCT (0.762) and the summary ROC curve location for N0 invasion using EUS was closer to the upper left corner than those using MDCT, which indicate the better diagnostic performance of EUS vs. MDCT (Figure 8).

##### N0 Stage

**Figure 9:**
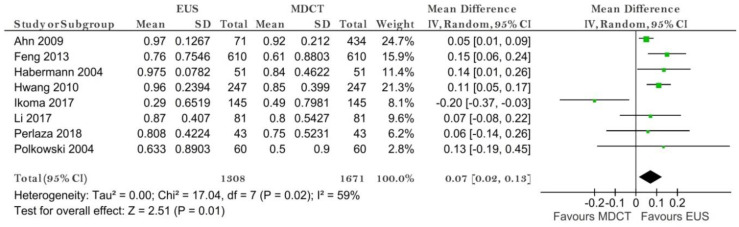
Forest plot of sensitivity for N0 staging.

Eight studies reporting data on 1671 patients were included for this test. The sensitivity value for EUS was significantly higher than for MDCT (*p* = 0.01) after using a random-effects model (χ^2^ = 17.04, I^2^ = 59%, *p* = 0.02) (Figure 9).

**Figure 10:**
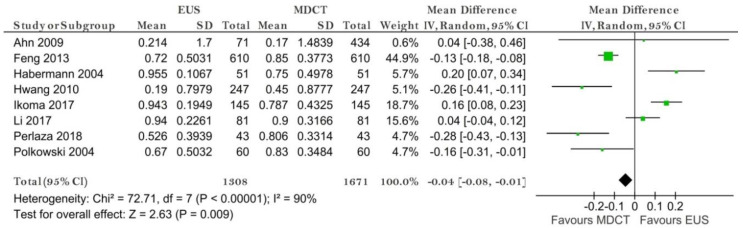
Forest plot of sensitivity for N0 staging.

The specificity value for EUS was significantly smaller than for MDCT (*p* = 0.009) (Figure 10).

**Figure 11:**
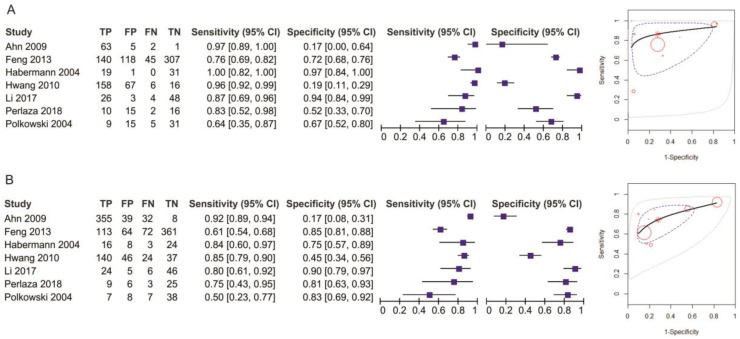
Pooled sensitivity and specificity for N0 staging of EUS (**A**) and MDCT (**B**).

The pooled sensitivity of EUS for the N0 stage was 0.815 (95%CI, 0.617–0.923), higher than that of MDCT, which was 0.732 (95%CI, 0.600–0.832). The pooled specificity values of EUS for the N0 stage was 0.699 (95%CI, 0.422–0.881), smaller than that of MDCT, which was 0.710 (95%CI, 0.517–0.849). The AUC for EUS (0.831) was bigger than for MDCT (0.779) and the summary ROC curve location for N0 invasion using EUS was closer to the upper left corner than those using MDCT, which indicate a better diagnostic performance by EUS than by MDCT (Figure 11).

##### N1 Stage

Six studies reporting data on 1092 patients were included for this test. The sensitivity and specificity values for EUS and MDCT were comparable (*p* = 0.68 and *p* = 0.98, respectively) after using a random-effects model for the high heterogeneity of the included studies (χ^2^ = 21.24, I^2^ = 77%, *p* = 0.0006).

The pooled sensitivity of EUS for the N1 stage was 0.446 (95%CI, 0.250–0.661), slightly smaller than that of MDCT, which was 0.488 (95%CI, 0.325–0.654). The pooled specificity values of EUS for the N1 stage was 0.805 (95%CI, 0.64–0.906), higher than that of MDCT, which was 0.749 (95%CI, 0.644–0.83). The AUC for EUS (0.69) was the same as the AUC for MDCT (0.693) and the summary ROC curve location for N1 invasion using EUS was the same as the upper left corner as those using MDCT, which indicate no difference between the diagnostic performance of EUS vs. MDCT.

##### N2 Stage

Six studies reporting data on 1092 patients were included for this test. The sensitivity value for MDCT was significantly higher than for EUS (*p* = 0.03) after using a random-effects model for the high heterogeneity of the included studies (χ^2^ = 12.77, I^2^ = 61%, *p* = 0.03). 

The specificity value for EUS was higher than for MDCT (*p* = 0.31) after using a random-effects model for the significant heterogeneity between studies (χ^2^ = 47.01, I^2^ = 89%, *p* < 0.00001).

The pooled sensitivity of EUS for the N2 stage was 0.301 (95%CI, 0.089–0.655), smaller than that of MDCT, which was 0.562 (95%CI, 0.406–0.707). The pooled specificity value of EUS for the N2 stage was 0.897 (95%CI, 0.805–0.948), almost the same as that of MDCT, which was 0.867 (95%CI, 0.726–0.941). The AUC for EUS (0.827) was slightly bigger than for MDCT (0.751) and the summary ROC curve location for N2 invasion using EUS was the same as the upper left corner as those using MDCT, which indicate no difference between the diagnostic performance of EUS vs. MDCT.

##### N3 Stage

Five studies reporting data on 1041 patients were included for this test. The sensitivity values were comparable (*p* = 0.74) after using a fixed-effects model for no heterogeneity of the included studies (χ^2^ = 4.40, I^2^ = 9%, *p* = 0.35). The specificity value for EUS was higher than that for MDCT (*p* = 0.06) after using a random-effects model for the significant heterogeneity between studies (χ^2^ = 12.26, I^2^ = 67%, *p* = 0.02).

The pooled sensitivity of EUS for the N3 stage was 0.162 (95%CI, 0.067–0.342), slightly smaller than that of MDCT, which was 0.211 (95%CI, 0.078–0.457). The pooled specificity value of EUS for the N3 stage was 0.989 (95%CI, 0.956–0.997), almost the same as that of MDCT, which was 0.967 (95%CI, 0.941–0.982). The AUC for EUS (0.712) was smaller than for MDCT (0.93), which indicates the better performance of MDCT for the N3 stage.

##### M Stage

**Figure 12:**
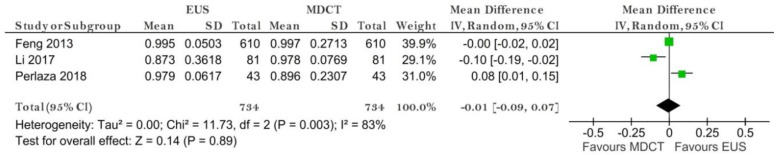
Forest plot of sensitivity for M staging.

Three studies reporting data on 734 patients were included for this test. The sensitivity value for EUS was the same as the one for MDCT (*p* = 0.89) after using a random-effects model for the high heterogeneity of the two included studies (χ^2^ = 11.73, I^2^ = 83%, *p* = 0.003) (Figure 12).

**Figure 13:**
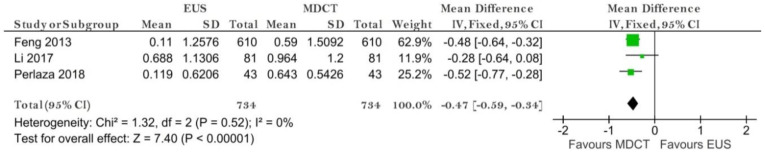
Forest plot of specificity for M staging.

The specificity value for MDCT was significantly higher than for EUS (*p* < 0.00001) after using a fixed-effects model for no heterogeneity between studies (χ^2^ = 1.32, I^2^ = 0%, *p* = 0.52) (Figure 13).

**Figure 14:**
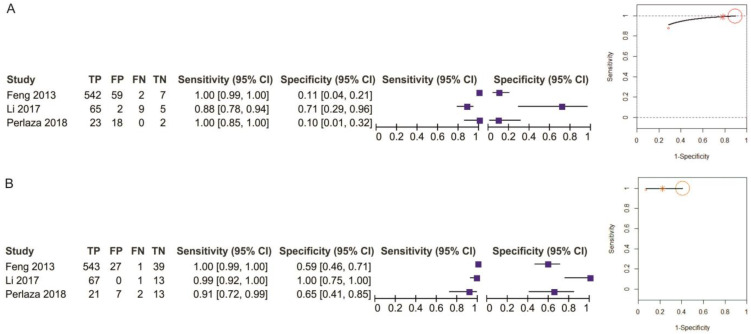
Pooled sensitivity and specificity for M staging of EUS (**A**) and MDCT (**B**).

The pooled sensitivity of EUS for the M stage was 0.980 (95%CI, 0.851–0.998), the same as the one of MDCT, which was 0.9800 (95%CI, 0.853–0.998). The pooled specificity value of EUS for the M stage was 0.252 (95%CI, 0.054–0.666), smaller than that of MDCT, which was 0.639 (95%CI, 0.524–0.739). The AUC for EUS (0.826) was higher than for MDCT (0.718) and the summary ROC curve location for M invasion using MDCT was closer to the upper left corner than those using EUS, which indicate the better diagnostic performance of MDCT than of EUS (Figure 14).

#### 3.3.2. EUS vs. EUS + MDCT

##### T1 Stage

**Figure 15:**
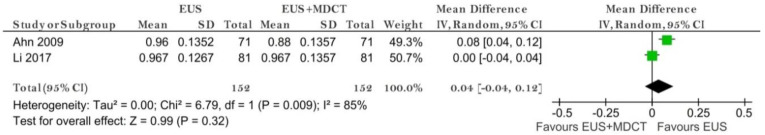
Forest plot of sensitivity for T1 staging in EUS vs. EUS + MDCT.

Two studies reporting data on 152 patients were included for this test. The sensitivity value for EUS was slightly higher, but not statistically significant, than for MDCT (*p* = 0.32) after using a random-effects model for the high heterogeneity of the included studies (χ^2^ = 6.79, I^2^ = 85%, *p* = 0.009) (Figure 15).

**Figure 16:**
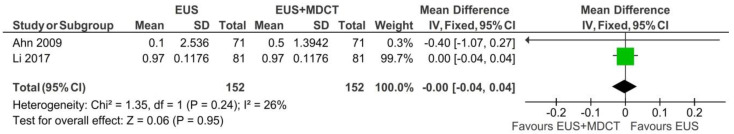
Forest plot of specificity for T1 staging in EUS vs. EUS + MDCT.

The specificity values for EUS and EUS + MDCT were comparable (*p* = 0.96) after using a fixed-effects model for no between-studies heterogeneity (χ^2^ = 1.35, I^2^ = 26%, *p* = 0.24) (Figure 16). 

**Figure 17:**
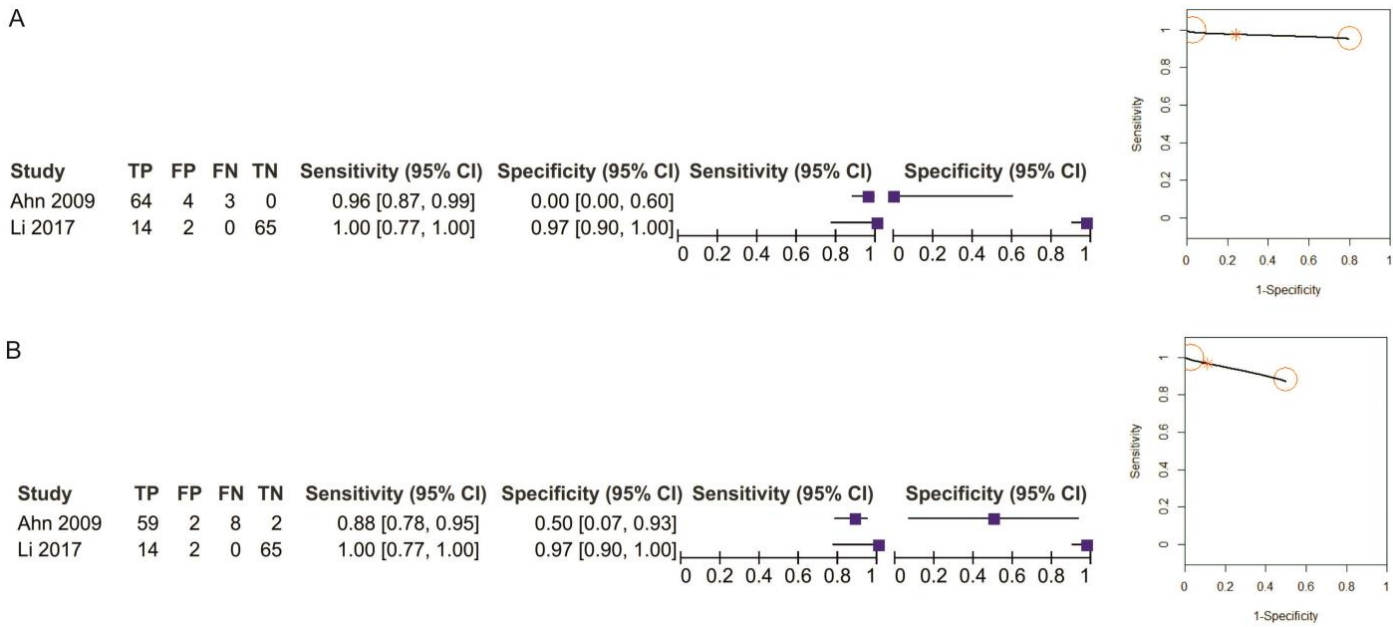
Pooled sensitivity and specificity for T1 staging of EUS (**A**) and EUS + MDCT (**B**).

The pooled sensitivity of EUS for the T1 stage was 0.958 (95%CI, 0.884–0.986), slightly higher than that of EUS + MDCT, which was 0.930 (95%CI, 0.742–0.984). The pooled specificity value of EUS for the T1 stage was 0.668 (95%CI, 0.01–0.998), smaller than that of EUS + MDCT, which was 0.844 (95%CI, 0.178–0.993). The AUC for EUS (0.95) and EUS + MDCT (0.93) are comparable and the summary ROC curve location for T1 invasion using EUS was the same to the upper left corner as those using EUS + MDCT (Figure 17).

## 4. Discussion

Gastric cancer requires precise and detailed imaging diagnosis to support curative surgery. Both early and advanced stages require a treatment strategy that refers to resection or pre-operative oncologic options [24]. While the original staging of a gastric tumor is best after pathologic analysis, ensuring a precise clinical stage is of major importance to maximize therapeutic management. EUS is considered the key technique for layer tumor distribution in gastric cancer, which makes it the most important tool for T assessment. On the other hand, CT, due to its wide distribution is the first imaging technique used in many institutions for the assessment of gastric cancer [25].

We performed a diagnostic meta-analysis of studies that included the diagnostic accuracies of EUS, MDCT and EUS+ MDCT for TNM stage assessment of gastric cancer (2047 patients). We compared the results from twelve studies, which were of a sufficiently high methodological quality to warrant highlighting the results, according to the QUADAS-2 analysis.

Our results suggest that EUS was superior to MDCT in preoperative T1 and N staging. No significant differences were observed in T2-T4 stages. While similar results were obtained in the meta-analysis of Nie et al. in 2017 [9], by the addition of four more studies we obtained different results for specificities for the N stages, with MDCT values being significantly higher than EUS values. Moreover, we performed the analysis separately for N1-N3 and also the M stage. We obtained comparable results for sensitivity and specificity for both EUS and MDCT for the N1 stage. However, from this point on, the sensitivity of MDCT was significantly higher than that of EUS for N2 (statistically significant) and N3 (not statistically significant) staging. Significant specificity of EUS vs. MDCT was demonstrated for N3 staging.

EUS is considered an important tool for T1 gastric cancer patients with ESD indication. However, when preparing for an ESD, preoperative imaging assessment is mandatory to establish if the tumor surpasses the T1b stage, thus the patient requires surgical resection. Even though the assessment of submucosal invasion is difficult, EUS remains the better option for imaging visualization [26]. For the T1 stage, we also had a group of patients (152) where we compared EUS and EUS + MDCT. Our results showed that even though there is a better accuracy in imaging diagnosis when using both techniques, sensitivity and specificity are not relevant. We did not obtain conclusive results for the T2-T4 stages. This could be related to several factors that may influence EUS accuracy, for example, an ulcerated tumor, size > 3 cm, histological type and mostly, location, with most of the tumors found within the antrum [27].

Over the years there have been much debate on preoperative gastric cancer staging, especially for T2 and T3, which require multimodality therapies [28]. Since the introduction of the AJCC Cancer staging manual [29], the use of EUS is highly recommended for the “Clinical stage” assessment. When comparing the N stage between the 2 techniques, MDCT scan proved to be more efficient for advanced stages. Even though most of the studies used different staging systems available at the time, we believed it was relevant to individualize the N stage. EUS is more reliable in predicting the presence of lymph nodes with a sensitivity of 84%, whereas MDCT showed a sensitivity of 75%. However, when separating the N stage, the sensitivity decreased progressively, whereas the specificity increased when reaching N3 stage. Thus, both diagnostic procedures might be necessary in advanced stages.

We also assessed the M stage, even though only three studies focused on gastric cancer metastases. MDCT had a better significant specificity than EUS, but no significant difference in sensitivity. It is important to note that imaging biomarkers may represent effective additional tools in the diagnosis and staging of gastric cancer. For example, MDCT-based texture features such as the apparent diffusion coefficient seem to be promising biomarkers for the evaluation of the aggressiveness (T and N stage), treatment response and prognosis of gastric cancer [30]. Furthermore, MDCT imaging biomarkers hold promise as prognostic factors, with potential for guiding treatment and follow-up strategy [31,32].

There are some limitations in the present diagnostic meta-analysis, including that meta-analyses can be a significant source of heterogeneity. We only had 12 studies that focused on EUS vs. CT, and most of them had a staging system according to their related time. For some outcomes (T1) there was significant heterogeneity of results across studies, but the use of the random-effects model partially mitigates this concern.

A potential further limitation of our study was the inclusion of retrospective studies. Despite these limitations, our study extends the findings of the previous meta-analysis with reference to T1-T4, N0-N3, M0-M1 invasion for EUS vs. CT and EUS vs. EUS + CT.

## 5. Conclusions

Both techniques are reliable tools for the preoperative assessment of gastric cancer. Our results support the use of EUS for the T1 stage, which can make a difference in response from ESD to multimodal therapy in gastric cancer patients. Even though EUS helped differentiate between the presence of invaded nodules, N stages should be carefully assessed by both methods since there is insufficient data.

## Figures and Tables

**Table 1:** Characteristics of included studies.

Study	Edition of TNM Classification	Study Type	No. of Patients	Age, Years	Gender	Location, n
Ahn 2009 [12]	The 6th UICC	P	434	Mean (SD) = 55.9 (11.6)	278 men 156 women	Upper = 39 Middle = 81 Lower = 311 Entire = 3
Cimavilla-Roman 2017 [13]	The 7th AJCC	R	42	Mean (SD) = 70.04 (12.36)	26 men 16 women	n/a
Fairweather 2015 [14]	The 7th AJCC	R	49	Median = 67 Range = 31–90	31 men 18 women	Antrum = 15 Cardia = 10
Feng 2013 [15]	The 6th UICC	R	610	Median = 57 Range = 22–84	482 men 128 women	Upper = 272 Middle = 93 Lower = 232 Entire = 13
Furukawa 2011 [16]	The 7th UICC	R	175	Mean (SD) = 66.3 (10.5)	133 men 42 women	Upper = 28 Middle = 94 Lower = 57 Entire = 7
Giganti 2016 [17]	The 7th UICC	P	52	Mean (SD) = 68.5 (1.35) Range: 43–85	33 men 19 women	Siewert II = 3 Siewert III = 4 Stomach = 45
Habermann 2004 [18]	n/a	R	51	Mean = 62 Range = 47–76	34 men 17 women	Fundus = 2 Body = 14 Antrum = 29 Pyloric region = 6
Hwang 2010 [19]	n/a	R	277	Mean = 53 IQR = 49–56	171 men 106 women	Cardia = 15 Body = 48 Angle = 24 Antrum = 46 Prepyloric = 8
Ikoma 2017 [20]	The 7th AJCC	R	145	<65 = 86 ≥ 65 = 101	106 men 81 women	Body = 60 Antrum = 88 Gastroesophageal junction = 23 Cardia = 16
Li 2017 [21]	The 5th UICC	P	81	Mean (SD) = 56.8 (11.51)	58 men 23 women	n/a
Perlaza 2018 [22]	The 7th IUAC	P	50 (-7 stenosis)	Mean (SD) = 65.7 (12.1)	30 men 20 women	Fundus = 7 Body = 21 Antrum = 22
Polkowski 2004 [23]	The 4th UICC	P	88	Mean = 63 IQR = 52.5–70	56 men 32 women	Upper third = 21 Upper + middle third = 21 Upper + middle + lower third = 6 Middle third = 13 Middle + lower third = 9 Lower third = 18

R, retrospective study; P, prospective study; n/a, not available; IQR, interquartile range.

**Table 2:** Sensitivity and specificity for endoscopic ultrasound (EUS) and multidetector computer tomography (MDCT) imaging to diagnose T, N and M staging.

	Sensitivity (%)			Specificity (%)		
	EUS Mean (95%CI)	MDCT Mean (95%CI)	Mean Difference (95%CI) *p*-Value	EUS Mean (95%CI)	MDCT Mean (95%CI)	Mean Difference (95%CI) *p*-Value
T1	71 (43, 88)	52 (26, 77)	0.24 (0.01, 0.47) *p* = 0.04	93 (75, 98)	94 (80, 98)	0.00 (−0.01, 0.01) *p* = 0.52
T2	67 (53, 79)	59 (40, 76)	0.06 (−0.21, 0.32) *p* = 0.67	83 (79, 87)	80 (73, 85)	0.03 (−0.03, 0.08) *p* = 0.32
T3	64 (49, 76)	63 (41, 82)	0.01 (−0.15, 0.17) *p* = 0.90	84 (75, 91)	81 (68, 89)	0.04 (−0.03, 0.12) *p* = 0.25
T4	52 (33, 70)	66 (46, 81)	−0.07 (−0.23, 0.09) *p* = 0.38	95 (87, 98)	96 (91, 98)	−0.01 (−0.03,0.02) *p* = 0.59
N0 vs. N1+	79 (64, 89)	73 (61, 82)	0.07 (0.01, 0.13) *p* = 0.02	64 (37, 84)	68 (53, 80)	−0.04 (−0.07, −0.01) *p* = 0.02
N0	82 (62, 92)	73 (60, 83)	0.07 (0.02, 0.13) *p* = 0.01	70 (42, 88)	71 (52, 85)	−0.04 (−0.08, −0.01) *p* = 0.009
N1	45 (25, 66)	49 (33, 65)	−0.05 (−0.26, 0.17) *p* = 0.68	80 (64, 91)	75 (64, 83)	0.00 (−0.07, 0.07) *p* = 0.98
N2	30 (9, 66)	56 (41, 71)	−0.21 (−0.39,−0.02) *p* = 0.03	90 (80, 95)	87 (73, 94)	0.05 (−0.04, 0.13) *p* = 0.31
N3	16 (7, 34)	21 (8, 46)	−0.02 (−0.12, 0.08) *p* = 0.74	99 (96, 99.9)	97 (94, 98)	0.02 (0.00, 0.03) *p* = 0.06
M	98 (85, 99)	98 (85, 99.8)	−0.01 (−0.09, 0.07) *p* = 0.89	25 (5, 67)	64 (52, 74)	−0.47 (−0.59,−0.34) *p* < 0.00001

**Table 3:** Sensitivity and specificity for EUS and EUS + MDCT imaging to diagnose T1 staging.

	Sensitivity (%)			Specificity (%)		
	EUS Mean (95%CI)	EUS + MDCT Mean (95%CI)	Mean Difference (95%CI) *p*-Value	EUS Mean (95%CI)	EUS + MDCT Mean (95%CI)	Mean Difference (95%CI) *p*-Value
T1	96 (88, 99)	93 (74, 98)	0.04 (−0.04, 0.12) *p* = 0.32	67 (1, 99)	84 (18, 99)	0.00 (−0.04, 0.04) *p* = 0.95

## Data Availability

The authors declare that the data of this research is available from the correspondence author on request.
